# Case Report: Fatal myocarditis and myasthenia gravis induced by immune checkpoint inhibitors: concurrent dual adverse events in an older adult

**DOI:** 10.3389/fonc.2025.1624369

**Published:** 2025-12-15

**Authors:** Omar Fakhreddine, Wassim Assaad, Nour El Meski, Firas Kreidieh, Samir Alam, Maurice Khoury

**Affiliations:** 1 Division of Cardiology, Department of Internal Medicine, American University of Beirut Medical Center, Beirut, Lebanon; 2 Department of Internal Medicine, American University of Beirut Medical Center, Beirut, Lebanon; 3 Division of Hematology-Oncology, Department of Internal Medicine, American University of Beirut Medical Center, Beirut, Lebanon

**Keywords:** immune checkpoint inhibitor, myocarditis, myasthenia gravis, renal cell carcinoma, immunotherapy

## Abstract

Immune checkpoint inhibitors (ICIs) have revolutionized the treatment of metastatic renal cell carcinoma (RCC), offering significant survival benefits. Their use, however, can come at the expense of severe immune-related adverse events (irAEs) involving the myocardium and nervous system. We report an 86-year-old male with relapsed clear cell RCC (Fuhrman grade 3, pT3aN0M0) who developed concurrent myocarditis and myasthenia gravis (MG) after receiving Pembrolizumab and Axitinib for multifocal mediastinal relapse. Despite early initiation of high-dose IV methylprednisolone within 24 hours, the patient’s condition deteriorated rapidly, resulting in pneumonia, acute kidney injury requiring hemodialysis, and death. This case highlights the fulminant course of ICI-induced myocarditis and MG, underscoring the importance of prompt recognition, immediate initiation of corticosteroids and IV immunoglobulin, and early consideration of second-line immunomodulatory therapies in severe cases.

## Introduction

Immune checkpoint inhibitors (ICIs) targeting CTLA-4, PD-1, and PD-L1 have revolutionized cancer therapy by enhancing anti-tumor T-cell activity (1). However, this immune activation can trigger immune-related adverse events (irAEs), which can affect virtually any organ system (2, 3). Although most irAEs are mild, myocarditis and myasthenia gravis (MG) are rare but often fatal complications (4–6). Overlap syndromes involving myocarditis, MG, and myositis have recently been described (7). Here, we present an elderly patient with recurrent high-grade clear cell RCC who developed fatal concurrent myocarditis and MG following Pembrolizumab and Axitinib therapy, highlighting diagnostic and therapeutic challenges.

## Case presentation

An 86-year-old male with dyslipidemia and benign prostatic hyperplasia was diagnosed in 2016 with clear cell RCC (Fuhrman grade 3, pT3aN0M0) and underwent right radical nephrectomy. In December 2023, surveillance CT revealed multiple enlarged mediastinal lymph nodes (right paratracheal and subcarinal, largest 2.5 cm), consistent with systemic relapse. Local therapy was deemed infeasible, and systemic therapy with Pembrolizumab (200 mg every 3 weeks) and Axitinib (5 mg twice daily) was initiated.

Twenty days after therapy initiation, he presented with dyspnea, diplopia, neck weakness, and dysphagia. Physical exam revealed left-sided ptosis and ophthalmoplegia. Labs showed CK 1,250 U/L (ref <200), Troponin I 0.345 ng/mL (ref <0.04), and elevated liver enzymes. ECG showed first-degree AV block with LBBB (HR 120 bpm). Echocardiogram demonstrated preserved ejection fraction.

Within 24 hours, he developed complete AV block (HR 30 bpm) requiring temporary pacing. Immunotherapy was stopped, and IV methylprednisolone (1 mg/kg/day) was initiated. Anti-AChR antibody was positive (4.9 nmol/L, ref <0.5); anti-MuSK was not measured. CT pulmonary angiogram excluded embolism; chest X-ray revealed right lower lobe pneumonia. Blood cultures were negative. Despite therapy, the patient’s condition deteriorated with respiratory failure and oliguric AKI requiring hemodialysis. He passed away despite maximal supportive care.

**Figure 1:**
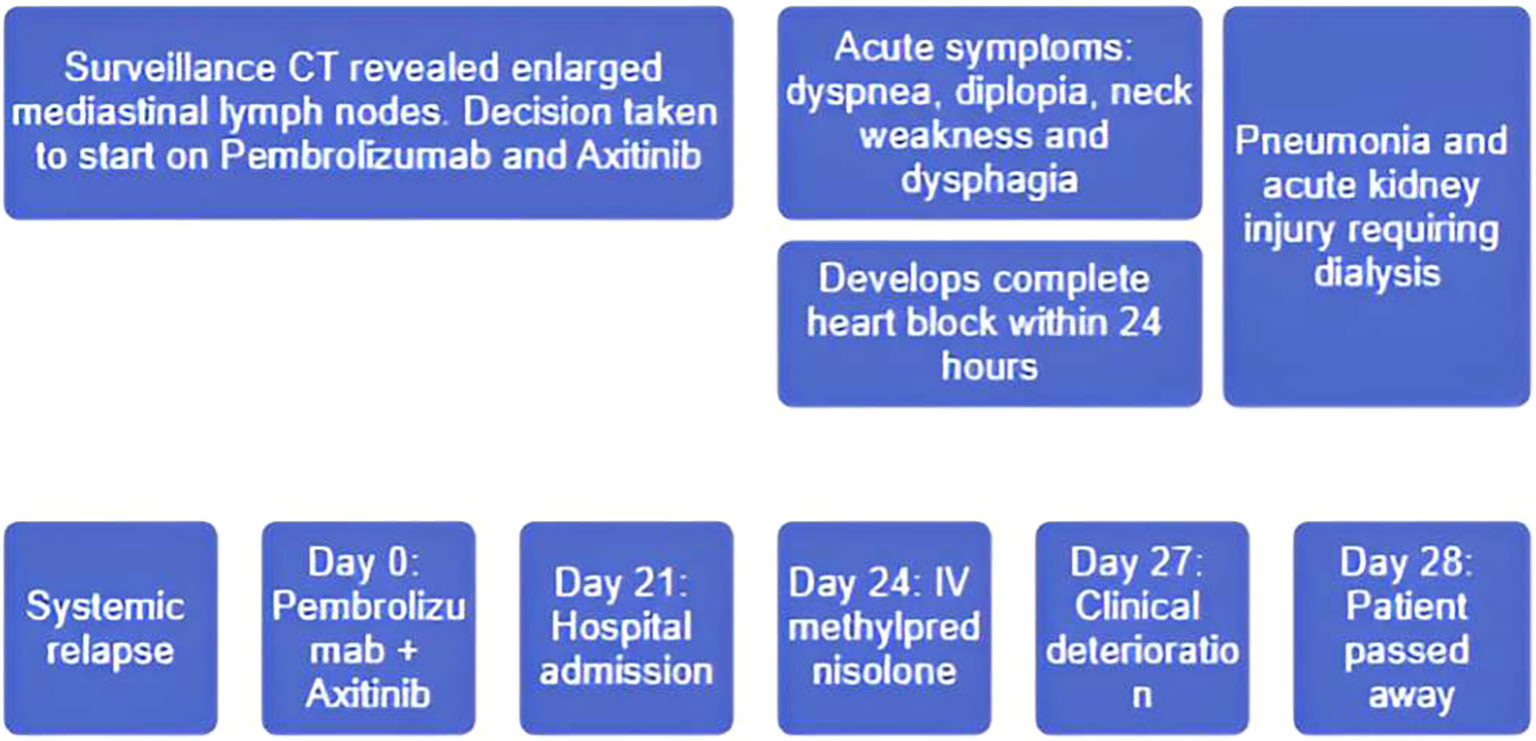
Clinical timeline. A schematic timeline illustrating the sequence of clinical events from the initiation of immunotherapy to the fatal outcome.

**Figure 1**. Clinical timeline summarizing the key clinical events: initiation of Pembrolizumab and Axitinib, symptom onset, hospital admission, cardiac conduction abnormalities, initiation of immunosuppressive therapy, development of complications, and eventual outcome.

## Discussion

Since its approval in 2021, pembrolizumab, an anti–PD-1 immune checkpoint inhibitor (ICI), has markedly transformed the treatment paradigm for renal cell carcinoma (RCC) (8). ICIs, alone or in combination with vascular endothelial growth factor inhibitors (VEGFis), have demonstrated durable responses and improved survival in patients with advanced or relapsed RCC (9). However, these benefits come with the risk of immune-related adverse events (irAEs), which can affect multiple organ systems, including the myocardium and neuromuscular junction (10).

The pathophysiology of irAEs remains incompletely understood. Proposed mechanisms include enhanced T-cell activation against shared antigens between tumor and host tissues, reduced peripheral tolerance due to PD-1/PD-L1 blockade, and cross-reactivity between cardiac or skeletal muscle epitopes and tumor neoantigens (3). Histopathological analyses often reveal T-lymphocytic infiltration of myocardial and skeletal muscle fibers, supporting an autoimmune-mediated process. Cytokine upregulation, particularly of IFN-γ and TNF-α, may further propagate tissue injury and inflammation (11).

Emerging evidence suggests that the combination of ICIs with VEGF inhibitors may increase the incidence of irAEs, including myocarditis. VEGF blockade modulates immune cell trafficking and enhances antigen presentation, which may amplify T-cell–mediated immune responses induced by PD-1 inhibition (12). However, the evidence remains limited and largely derived from retrospective analyses. Prospective studies are needed to confirm this association and delineate the underlying mechanisms. Our patient had no prior autoimmune disease, yet the concurrent use of pembrolizumab and axitinib may have contributed to the synergistic immune activation leading to fulminant myocarditis and myasthenia gravis.

ICI-induced myocarditis is rare, with an estimated incidence of 0.6–1.14% of treated patients, but carries a high mortality rate of up to 50% (13). Complete heart block (CHB) represents one of the most severe and life-threatening presentations. In multicenter cohorts, CHB occurred in approximately 15–20% of ICI-myocarditis cases and was strongly associated with poor outcomes (14). Several case reports mirror our observation of pembrolizumab-induced myocarditis presenting with CHB and myasthenia overlap, highlighting the fulminant and often fatal nature of this syndrome (15).

The diagnosis of ICI-related myocarditis remains challenging due to overlapping features with ischemic or infectious etiologies. Troponin elevation, new conduction abnormalities, and arrhythmias should prompt early evaluation. While echocardiography and ECG lack specificity, cardiac magnetic resonance (CMR) remains the preferred noninvasive diagnostic tool (16). Unfortunately, CMR was contraindicated in our patient due to the presence of a transvenous pacemaker.

The notion of screening older adults for underlying cardiac disease prior to ICI initiation is conceptually appealing but not currently evidence-based. Although baseline ECG and troponin assessment may help identify patients at risk of subclinical cardiac disease, there is no established evidence that such screening prevents the occurrence of ICI-related myocarditis (17). Nonetheless, given the potentially fatal consequences, obtaining baseline cardiac biomarkers and ECG before initiating therapy, especially in elderly or high-risk individuals, may be a pragmatic approach for early detection rather than prevention.

Prompt recognition and discontinuation of immunotherapy are critical once myocarditis or neuromuscular irAEs are suspected. High-dose corticosteroids remain the cornerstone of treatment. Guidelines recommend methylprednisolone 1–2 mg/kg/day intravenously (or 1 g/day for severe myocarditis), followed by a slow taper over at least 4–6 weeks depending on clinical response. Early administration (within 24 hours) has been associated with improved outcomes and reduced major adverse cardiovascular events (18).

For steroid-refractory cases, additional immunomodulatory agents have been reported to improve outcomes (19). In a large systematic review of ICI-related myasthenia gravis, patients treated up-front with IVIG or plasmapheresis (PLEX) achieved a 95% symptom-improvement rate compared to 63% with steroids alone (20). A case series of ten patients with overlapping myasthenia gravis, myositis and myocarditis reported that eight patients received intravenous immunoglobulin along with high-dose methylprednisolone and nine of the ten survived to discharge, suggesting a clear benefit of early multimodal immunosuppression (21). Moreover, a more recent observational series further highlights that in older adults with overlap syndromes, treatment should escalate rapidly to include IVIG, plasmapheresis or other immunomodulators alongside steroids given the high risk of rapid deterioration (22). Accordingly, in cases of suspected myocarditis with neuromuscular irAEs, clinicians should consider immediate initiation of adjunctive therapies especially IVIG and PLEX rather than relying on steroids alone, to optimize outcomes in this high-mortality setting.

Rechallenge with ICI therapy after resolution of myocarditis or severe neuromuscular irAEs is generally not recommended, given the high risk of recurrence and mortality. However, in select cases of mild myocarditis with complete recovery, rechallenge under close multidisciplinary monitoring may be considered on a case-by-case basis (23).

## Conclusion

This case highlights the potential severity of immune-related adverse events following combined pembrolizumab and axitinib therapy, particularly the rare overlap of myocarditis and myasthenia gravis presenting with complete heart block. It underscores the need for high clinical vigilance and early recognition of cardiac or neuromuscular symptoms in patients receiving immune checkpoint inhibitors, especially when used in combination with VEGF inhibitors. Multidisciplinary management, including prompt immunotherapy discontinuation and early initiation of high-dose corticosteroids, remains crucial to improving outcomes. Further research is needed to elucidate the mechanisms underlying combination therapy–related toxicity, to identify predictive risk factors, and to establish evidence-based guidelines for screening, treatment duration, and safe rechallenge strategies.

## Data Availability

The original contributions presented in the study are included in the article/supplementary material. Further inquiries can be directed to the corresponding author.
